# Use of artificial intelligence for sepsis risk prediction after flexible ureteroscopy: a systematic review

**DOI:** 10.1590/0100-6991e-20233561-en

**Published:** 2023-06-22

**Authors:** BEATRIZ MESALIRA ALVES, MIKHAEL BELKOVSKY, CARLO CAMARGO PASSEROTTI, EVERSON LUIZ DE ALMEIDA ARTIFON, JOSÉ PINHATA OTOCH, JOSÉ ARNALDO SHIOMI DA CRUZ

**Affiliations:** 1 - Universidade Nove de Julho, - São Bernardo do Campo - SP - Brasil; 2 - Universidade de São Paulo, Faculdade de Medicina - São Paulo - SP - Brasil; 3 - Hospital Alemão Oswaldo Cruz - São Paulo - SP - Brasil

**Keywords:** Ureteroscopy, Sepsis, Artificial Intelligence, Nephrolithiasis, Machine Learning, Ureteroscopia, Sepse, Inteligência Artificial, Nefrolitíase, Aprendizado de Máquina

## Abstract

**Introduction:**

flexible ureteroscopy is a minimally invasive surgical technique used for the treatment of renal lithiasis. Postoperative urosepsis is a rare but potentially fatal complication. Traditional models used to predict the risk of this condition have limited accuracy, while models based on artificial intelligence are more promising. The objective of this study is to carry out a systematic review regarding the use of artificial intelligence to detect the risk of sepsis in patients with renal lithiasis undergoing flexible ureteroscopy.

**Methods:**

the literature review is in accordance with the Preferred Reporting Items for Systematic Reviews and Meta-Analysis (PRISMA). The keyword search was performed in MEDLINE, Embase, Web of Science and Scopus and resulted in a total of 2,496 articles, of which 2 met the inclusion criteria.

**Results:**

both studies used artificial intelligence models to predict the risk of sepsis after flexible uteroscopy. The first had a sample of 114 patients and was based on clinical and laboratory parameters. The second had an initial sample of 132 patients and was based on preoperative computed tomography images. Both obtained good measurements of Area Under the Curve (AUC), sensitivity and specificity, demonstrating good performance.

**Conclusion:**

artificial intelligence provides multiple effective strategies for sepsis risk stratification in patients undergoing urological procedures for renal lithiasis, although further studies are needed.

## RESUMO

**Introdução:**

a ureteroscopia flexível é uma técnica cirúrgica minimamente invasiva utilizada para o tratamento de litíase renal. A urosepse pós-operatória é uma complicação rara, mas potencialmente fatal. Os modelos tradicionais utilizados para prever o risco dessa condição apresentam precisão limitada, enquanto modelos baseados em inteligência artificial são mais promissores. O objetivo desse estudo é realizar uma revisão sistemática a respeito do uso de inteligência artificial para detecção do risco de sepse em pacientes com litíase renal submetidos à ureteroscopia flexível.

**Métodos:**

a revisão de literatura está de acordo com o Preferred Reporting Items for Systematic Reviews and Meta-Analysis (PRISMA). A busca com palavras-chave foi realizada no MEDLINE, Embase, Web of Science e Scopus e resultou no total de 2.496 artigos, dos quais 2 se enquadraram nos critérios de inclusão.

**Resultados:**

os dois estudos utilizaram modelos de inteligência artificial para predizer o risco de sepse após utereroscopia flexível. O primeiro teve uma amostra de 114 pacientes e foi baseado em parâmetros clínicos e laboratoriais. O segundo teve uma amostra inicial de 132 pacientes e foi baseado em imagens de tomografia computadorizada no pré-operatório. Ambos obtiveram boas medidas de Area Under the Curve (AUC), sensibilidade e especificidade, demonstrando boa performance.

**Conclusão:**

a inteligência artificial fornece múltiplas estratégias eficazes para estratificação do risco de sepse em pacientes submetidos a procedimentos urológicos para litíase renal, ainda que mais estudos sejam necessários.

## INTRODUCTION

Renal lithiasis is a disease with increasing prevalence in recent years and has both non-surgical and surgical treatments^1^. Flexible ureteroscopy is a minimally invasive surgical technique, widely used not only for treatment, but also for the diagnosis of urological conditions^2^. Although its complication rates are relatively low, the procedure can result in postoperative urosepsis, a serious and potentially fatal infection^3^. Thus, timely detection and adequate management are crucial to prevent its progression to septic shock, multiple organ failure, and ultimately death^4^.

Since sepsis is a systemic inflammatory response associated with organ dysfunction due to an infection, it includes signs and symptoms such as fever, tachypnea, tachycardia, and arterial hypotension^5^. Screening can be performed using the Sequential Organ Failure Assessment (SOFA) and Quick Sequential Organ Failure Assessment (qSOFA) scores, with the diagnosis given by an increase of 2 or more points in SOFA and suspected or confirmed infection^6^.

Several clinical and laboratory parameters have been identified as risk factors for postoperative sepsis^3^. Traditional risk prediction models based on these parameters have shown limited accuracy, leading to a growing interest in the development of algorithms based on artificial intelligence to predict disease risk after urological procedures^7^. These algorithms can analyze large volumes of data from electronic health records, including vital signs, laboratory values, and clinical history.

Recent advances in artificial intelligence have brought new opportunities for the creation of models based on risk factors, enabling strategies to improve clinical outcomes and minimize postoperative morbidity. The studies carried out reported promising results, with artificial intelligence algorithms demonstrating greater accuracy than traditional models for risk prediction^5^ ^,^ ^8^. However, the implementation of such tools in clinical practice still faces several challenges, for example data quality and privacy concerns, transparency and interpretability of algorithms, integration with clinical workflows, and costs.

The purpose of this article is to provide an overview of the current state of knowledge about using artificial intelligence to predict sepsis risk after ureteroscopy for kidney stones and to discuss the challenges and opportunities for bringing these tools into patient care.

## METHODS

We conducted this systematic review from October to November 2022, registered in the Prospectve Register of Systematic Reviews (PROSPERO - 42022374866), and in accordance with the Preferred Reporting Items for Systematic Reviews and Meta-Analysis (PRISMA) checklist. We performed the search strategy according to the PICO criteria (Patient, Intervention, Comparison, and Outcome), where P: patients with renal lithiasis treated with flexible ureteroscopy, I: machine learning models, C: no models, and O: postoperative complications. The databases used were MEDLINE, Embase, Web of Science, and Scopus, with the combination of keywords: “uretero*”, “renoscopy”, “fURS” (flexible ureteroscopy), “RIRS” (retrograde intrarenal surgery), “retrograde intrarenal surgery”, “deep learning”, “machine learning”, “artificial neural network”, “artificial intelligence”, without a defined search period.

The inclusion criteria were:

1. Studies of patients with renal lithiasis treated with flexible ureteroscopy involving machine learning models; and
2. Studies in English.

The exclusion criteria were:

1. Editorials, comments, summaries, reviews, or book chapters; and
2. Studies in animals, laboratories, or cadavers.

We exported all articles to the EndNote software. First, we evaluated the titles, then the abstracts, and, after screening, we analyzed their full texts to select those that met the inclusion criteria.

## RESULTS

**Figure 1:**
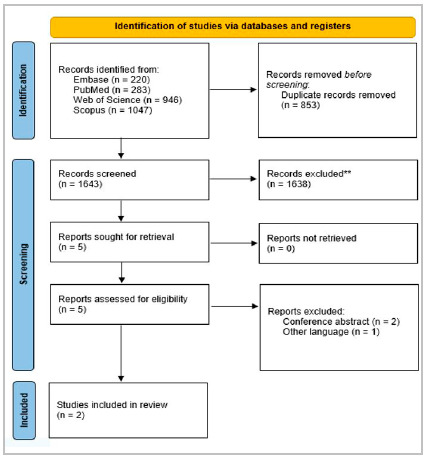
Preferred Reporting Items for Systematic Reviews and Meta-Analyses (PRISMA).

We initially selected 2,496 and, after screening according to the inclusion criteria, two articles were included in the final review (Figure 1).

In the first study, Pietropaolo et al.^5^ used a sample of 114 patients to analyze the use of a predictive machine learning model in patients who had urosepsis and needed support in the Intensive Care Unit (ICU) after ureteroscopy. Of the 114 patients, 57 developed urosepsis (group A) and 57 did not (group B).

The machine learning model implemented was the randomforests package of the R statistics software. The predicted risk of having sepsis was 82% in group A and the predicted risk of not having sepsis was 80% in group B. Model accuracy was 81.3% (95% CI 63.7 92.8%), sensitivity = 0.80, specificity = 0.82, and Area Under the Curve (AUC) = 0.89. Variables such as the proximal location of the calculus, prolonged stent use, large calculus size, and long operative time were significant for the occurrence of the disease^5^. 

In the second study, Chen et al.^8^ investigated models to assess the risk of sepsis after calculus removal from the analysis of preoperative computed tomography images. Each model was developed on an initial sample of 132 patients (44 patients who had sepsis and 88 who did not), matched for preoperative demographic characteristics, and then validated in a group of 40 patients. Female sex, presence of fever, and positive preoperative urine culture were significant risk factors for the development of urosepsis in the univariate analysis and were equalized in both groups after the matching process. 

The first model was the Least Absolute Shrinkage and Selection Operator (LASSO) and obtained an AUC = 0.881 (95% CI, 0.813-0.931), with a sensitivity of 79.55% and specificity of 96.59%. When the developed model was tested in the validation group, it continued to perform well, with AUC = 0.783 (95% CI, 0.766-0.801) and sensitivity and specificity of 88%. The second model was a Deep Neural Network (DNN), displaying an AUC = 0.920 (95% CI, 0.906-0.933) in the internal validation, with a sensitivity of 85.71% and specificity of 94.73%. When the developed model was tested in the validation group, it continued to perform well, with AUC = 0.874 (95% CI, 0.856-0.891), sensitivity of 77%, and specificity of 88.67%^8^.

## DISCUSSION

The worldwide increase in the prevalence of renal lithiasis is directly related to the increase in obesity and diabetes. The general recommendations for adequate control of comorbidities, increased fluid intake, decreased salt intake, and moderate protein consumption are maintained^1^. Drug and surgical treatments depend on factors such as the size of the kidney stones^9^.

According to the guidelines of the European Association of Urology, flexible ureteroscopy is the main surgical treatment for renal calculi smaller than 20mm^10^ and presents high calculi free rates, 90% for the ones smaller than 10mm and 80% for those smaller than 15mm, especially when compared with percutaneous nephrolithotomy rates for stones of the same size^9^.

Although being a minimally invasive surgery, it can present complications due to urinary tract infections. A recent systematic review found that the sepsis rate ranged from 0.5% to 11.1% and the septic shock rate ranged from 0.3% to 4.6%^11^. The occurrence of sepsis after flexible ureteroscopy has, as risk factors, presence of comorbidities, age below 40 years, positive urine culture^11^, anatomical anomalies of the urinary tract^12^, female sex^13^, prolonged surgical time^14^, larger stones, high irrigation pressure^15^, and insertion of a double-J catheter after the procedure^16^.

Preoperative identification of patients at higher risk of developing postoperative urosepsis can help create preventive strategies, such as prophylactic antibiotic therapy, preoperative counseling, and intraoperative support, in addition to avoiding unnecessary antibiotic therapy in low-risk patients. Such measures would result in a better prognosis^5^.

The use of artificial intelligence is expanding every day due to the ability of a machine to perform human cognitive tasks and thus bring many benefits to the areas of activity. Machine learning, deep learning and artificial neural network are some of its strands, and the function of the first is to allow the computer to recognize patterns and create predictions through algorithms, building a learning model^5^. Within medicine, specifically urology, the application of technology assists in diagnosis, detection of the composition of kidney stones and prediction of treatment results, including complications and recurrence rate^17^.

Through the articles selected in our review, an important advance in the area was identified, with the objective of predicting an individualized prognosis of the risk of sepsis after flexible ureteroscopy. The radiomic model by Chen et al. proposes to predict the risk of sepsis after ureteroscopy only using tomographic images^8^, while the study by Pietropaolo et al. performs a more conventional approach, performing a multivariate analysis of clinical variables that meets the univariate results previously demonstrated in the literature^5^.

Parallel to the model by Chen et al.^8^, Blum et al.^18^ created a machine learning structure to improve the early detection of hydronephrosis due to obstruction of the pelvic-ureteral junction based on image data and obtained an accuracy of 93% in cases in need of surgery. Kocak et al.^19^ developed a machine learning model based on computed tomography results to distinguish three main subtypes of Renal Cell Carcinoma (RCC). The model was able to satisfactorily distinguish non-RCC from RCC. Feng et al.^20^ used a machine learning approach to differentiate small sizes (<4cm) of angiomyolipomas and carcinoma on computed tomography scans with high accuracy, sensitivity and specificity.

Likewise, corresponding to the model by Pietropaolo et al.^5^, Song et al.^7^ evaluated in their review whether machine learning models were superior compared to logistic regression, which is a more conventional forecasting model. Both techniques were used in the prediction of acute kidney injury and it was concluded that, in the literature, machine learning was superior due to greater adaptability. Aminsharifi et al.^21^ analyzed data from 146 adult patients undergoing percutaneous nephrolithotomy to validate the efficiency of a machine learning model to predict outcomes after the procedure. The program predicted surgical outcomes with an accuracy of up to 95%.

Even with the diversity of research in the area, our study observed few articles that specifically analyzed surgical results of flexible ureteroscopy involving artificial intelligence. A possible explanation for this is that, despite being a very versatile technology, the development of artificial intelligence models requires technical knowledge that is not accessible to most centers, either due to complexity, lack of incentive, among others. Also, internal and external validations must be conducted to confirm the accuracy and reliability of the models, reducing biases. However, once developed, we expect these models to integrate multiple approaches in the development of personalized medicine.

## CONCLUSION

Stratification of the risk of sepsis is fundamental for the operative planning of patients undergoing urological procedures in renal lithiasis, to guarantee the vitality of the patient. The literature review showed that artificial intelligence provides multiple effective strategies for this purpose, although further studies are needed.

## References

[1] Sorokin I, Mamoulakis C, Miyazawa K, Rodgers A, Talati J, Lotan Y (2017). Epidemiology of stone disease across the world. World J Urol.

[2] Doizi S, Traxer O (2018). Flexible ureteroscopy technique, tips and tricks. Urolithiasis.

[3] Blackmur JP, Maitra NU, Marri RR, Housami F, Malki M, Mcilhenny C (2016). Analysis of factors' association with risk of postoperative urosepsis in patients undergoing ureteroscopy for treatment of stone disease. J Endourol.

[4] Gavelli F, Castello LM, Avanzi GC (2021). Management of sepsis and septic shock in the emergency department. Intern Emerg Med.

[5] Pietropaolo A, Geraghty RM, Veeratterapillay R, Rogers A, Kallidonis P, Villa L (2021). A machine learning predictive model for post-ureteroscopy urosepsis needing intensive care unit admission a case-control yau endourology study from nine european centres. J Clin Med.

[6] Font MD, Thyagarajan B, Khanna AK (2020). Sepsis and Septic Shock - Basics of diagnosis, pathophysiology and clinical decision making. Med Clin North Am.

[7] Song X, Liu X, Liu F, Wang C (2021). Comparison of machine learning and logistic regression models in predicting acute kidney injury A systematic review and meta-analysis. Int J Med Inform.

[8] Chen M, Yang J, Lu J, Zhou Z, Huang K, Zhang S (2022). Ureteral calculi lithotripsy for single ureteral calculi can DNN-assisted model help preoperatively predict risk factors for sepsis?. Eur Radiol.

[9] Skolarikos A, Gross AJ, Krebs A, Unal D, Bercowsky E, Eltahawy E (2015). Outcomes of flexible ureterorenoscopy for solitary renal stones in the CROES URS global study. J Urol.

[10] Bozzini G, Filippi B, Alriyalat S, Calori A, Besana U, Mueller A (2021). Disposable versus reusable ureteroscopes a prospective multicenter randomized comparison. Res Rep Urol.

[11] Corrales M, Sierra A, Doizi S, Traxer O (2022). Risk of sepsis in retrograde intrarenal surgery a systematic review of the literature. Eur Urol Open Sci.

[12] Ozgor F, Sahan M, Cubuk A, Ortac M, Ayranci A, Sarilar O (2019). Factors affecting infectious complications following flexible ureterorenoscopy. Urolithiasis.

[13] Nevo A, Mano R, Baniel J, Lifshitz DA (2017). Ureteric stent dwelling time a risk factor for post-ureteroscopy sepsis. BJU Int.

[14] Sugihara T, Yasunaga H, Horiguchi H, Nishimatsu H, Kume H, Ohe K (2013). A nomogram predicting severe adverse events after ureteroscopic lithotripsy 12,372 patients in a Japanese national series. BJU Int.

[15] Hu W, Zhou PH, Wang W, Zhang L, Zhang Bin X (2016). Prognostic value of adrenomedullin and natriuretic peptides in uroseptic patients induced by ureteroscopy. Mediators Inflamm.

[16] Ogreden E, Oguz U, Demirelli E, Benli E, Özen Ö (2018). The impact of ureteral Double-J stent insertion following ureterorenoscopy in patients with ureteral stones accompanied by perirenal fat stranding. Arch Ital Urol Androl.

[17] Hameed BMZ, Shah M, Naik N, Rai BP, Karimi H, Rice P (2021). The Ascent of Artificial Intelligence in Endourology a Systematic Review Over the Last 2 Decades. Curr Urol Rep.

[18] Blum ES, Porras AR, Biggs E, Tabrizi PR, Sussman RD, Sprague BM (2018). Early Detection of Ureteropelvic Junction Obstruction Using Signal Analysis and Machine Learning A Dynamic Solution to a Dynamic Problem. J Urol.

[19] Kocak B, Yardimci AH, Bektas CT, Turkcanoglu MH, Erdim C, Yucetas U (2018). Textural differences between renal cell carcinoma subtypes Machine learning-based quantitative computed tomography texture analysis with independent external validation. Eur J Radiol.

[20] Feng Z, Rong P, Cao P, Zhou Q, Zhu W, Yan Z (2018). Machine learning-based quantitative texture analysis of CT images of small renal masses Differentiation of angiomyolipoma without visible fat from renal cell carcinoma. Eur Radiol.

[21] Aminsharifi A, Irani D, Tayebi S, Jafari Kafash T, Shabanian T, Parsaei H (2020). Predicting the Postoperative Outcome of Percutaneous Nephrolithotomy with Machine Learning System Software Validation and Comparative Analysis with Guy's Stone Score and the CROES Nomogram. J Endourol.

